# Beta-amyloid imaging with florbetaben

**DOI:** 10.1007/s40336-015-0102-6

**Published:** 2015-02-12

**Authors:** Osama Sabri, John Seibyl, Christopher Rowe, Henryk Barthel

**Affiliations:** 1 Leipzig University, Leipzig, Germany; 2 Institute of Neurodegenerative Disorders, New Haven, CT USA; 3 University of Melbourne, Heidelberg, Australia

**Keywords:** PET, Beta-amyloid, Alzheimer, Florbetaben

## Abstract

Florbetaben is a fluorine-18 (^18^F)-labeled stilbene derivative that was developed as a positron emission tomography (PET) tracer for routine clinical application to visualize β-amyloid plaques in the Alzheimer’s disease (AD) brain. The tracer successfully completed a global multicenter phase 0–III development program and was, as a consequence, recently approved by the US Food and Drug Administration and the European Medicines Agency. This review provides an overview on the florbetaben tracer characteristics and preclinical data leading to its human testing. Further, the favorable results of human pharmacokinetics, safety, and dosimetry evaluation of florbetaben are presented. Next, the results of the clinical testing of florbetaben are discussed, in which the tracer was shown to sensitively and specifically detect β-amyloid neuritic plaques, as evidenced by employing different gold standards (from clinical diagnosis to post mortem histopathology). The potential of florbetaben to predict AD dementia in cases of mild cognitive impairment and to assist in the differential diagnosis in cases of dementia is also described. Finally, potential clinical impact and clinical routine PET image acquisition and analysis protocols for florbetaben are discussed. Taken together, the evidence shows that florbetaben is a valuable β-amyloid-targeting PET tracer in the clinic with great potential to serve as a biomarker supporting clinical AD diagnosis.

## Introduction

The deposition of β-amyloid is considered one of the initial events in the pathogenesis of Alzheimer’s disease (AD) [1], and most likely begins years before the initial onset of detectable cognitive symptoms, followed by gradual progression [2]. So far, however, cerebral β-amyloid plaques are only detectable in postmortem histopathology, which excludes this approach from establishment as a diagnostic tool during lifetime in AD patients.

Instead, clinical testing is still regarded as the standard tool to diagnose AD. However, especially at the prodromal AD stage of mild cognitive impairment (MCI) and even at the dementia stage of this disease, clinical testing lacks accuracy to establish a reliable AD diagnosis [3, 4]. This notion has prompted incorporating biomarkers as add-on to clinical testing into the AD diagnostic workflow [5, 6]. In this regard, the development of radiotracers to visualize β-amyloid plaques in vivo in the human brain is an important and active area of radiopharmaceutical research. β-Amyloid imaging agents not only are useful for early AD diagnosis [7], but also improve our understanding of the neuropathogenesis of AD and other neurodegenerative disorders [8]. Further, the current development of β-amyloid-targeted, disease-modifying therapies underscores the need for imaging biomarkers, as they may facilitate efficacy evaluation of the agent in question [9], as well as identifying patients who would benefit from therapy [10].

The first experimental positron emission tomography (PET) tracer used clinically for visual and quantitative assessment of β-amyloid deposition in the brain was 2-(1-(6-[(2-[^18^F]fluoroethyl)(methyl)amino]-2-naphthyl)ethylidene)malononitrile ([^18^F]FDDNP) [11]. Subsequently, the carbon-11 [^11^C]-labeled “thioflavin T” derivative, *N*-methyl-[^11^C]2-(4-methylaminophenyl)-6-hydroxybenzothiazole, also known as the [^11^C]Pittsburg B compound or [^11^C]PiB, was developed [7]. However, the 20-min radioactive half-life of ^11^C restricts the use of [^11^C]PiB to centers with an onsite cyclotron and extensive radiochemistry infrastructure. A fluorine-18 [^18^F]-labeled molecule with a radioactive half-life of 110 min is preferable, as it allows widespread distribution to PET centers without cyclotron, as is currently the case with [^18^F]fluorodeoxyglucose.

Florbetaben is an ^18^F-labeled polyethylene glycol stilbene derivative with high in vitro affinity and specificity for β-amyloid plaques. A comprehensive clinical development program was performed including a pivotal phase III study (for histological confirmation) and the pooled read of images, derived from the different clinical studies, as well as clinical safety and supportive efficacy data generated in individuals with Down’s syndrome (DS), AD dementia, other dementias, MCI, and in healthy individuals of varying ages. These studies generated a dataset on which the overall safety of florbetaben and its diagnostic accuracy in detecting neuritic β-amyloid plaques in the brain were substantiated.

This review summarizes and discusses the currently available knowledge on florbetaben.

## Florbetaben tracer characteristics and preclinical data

**Fig. 1:**
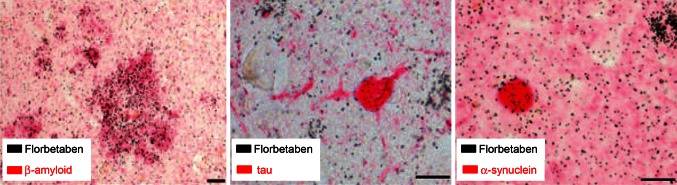
Specificity of florbetaben binding. Florbetaben binds specifically to β-amyloid plaques. High-resolution emulsion autoradiography using [^3^H]florbetaben (*black*) together with immunohistochemical staining for β-amyloid (*left*), tau (*middle*), and α-synuclein (*right*, *each colored in* *red*) in postmortem human brain slices. Co-localization of the florbetaben signal only with aggregated β-amyloid, but not with tau- or α-synuclein. *Scale bars* 15 μm. Reprinted from Ref. [13], with permission from Elsevier/Society of Radiopharmaceutical Sciences

In vitro, florbetaben shows nanomolar binding affinity to synthetic β-amyloid fibrils and to AD brain homogenate [12]. In addition, binding to β-amyloid plaques in postmortem AD brain sections was demonstrated by autoradiography, supported by immunohistochemistry and Bielschowsky staining. Florbetaben does not bind to tau- or α-synuclein pathology in human AD, dementia with Lewy bodies (DLB) or frontotemporal dementia brain tissue, demonstrating its specificity for β-amyloid deposits (Fig. 1) [13].

Several studies using human biomaterials were carried out with radiolabeled florbetaben (tritiated [^3^H], ^14^C, and ^18^F) or the non-radioactive ^19^F-derivative. Protein-binding studies were performed with non-radioactive drug substance in human serum; metabolic stability was tested by incubation of florbetaben with human plasma and with human liver microsomes; and human cytochrome P450 (CYP) isoforms involved in the in vitro metabolism of ^14^C-florbetaben were investigated and the inhibitory potency of florbetaben toward human CYP isoforms was tested [personal communication (Piramal)].

^18^F-florbetaben was found to be highly bound to plasma proteins (unbound fraction, 1.6 %), and it is metabolized by several CYP enzymes. Enzyme CYP4F2 predominantly mediates N-demethylation, whereas CYP2J2 and CYP3A4 contribute predominantly to the formation of polar metabolites. Among those, CYP2J2 and CYP4F2 were identified as the main enzymes involved in the metabolism of florbetaben. No risk of interactions with CYP enzymes was identified. Taken together, this preclinical evaluation indicates that ^18^F-florbetaben is a promising drug for imaging β-amyloid plaques typical of AD, as it was shown to bind to these structures in the brain and has a suitable pharmacokinetic (PK) profile [personal communication (Piramal)].

Florbetaben was also shown to be useful in brain β-amyloid imaging of small experimental animals. In a mouse model overexpressing Swedish mutant β-amyloid precursor protein (APP-Swe), ^18^F-florbetaben uptake was similar to that in wild-type (WT) mice at 10 and 13 months, but was significantly increased versus baseline at 16 months (+7.9 %; *p* < 0.01) and 20 months (+16.6 %; *p* < 0.001) [14]. There were no temporal changes in uptake from baseline in WT mice. In a follow-up study, the same authors demonstrated that applying a dedicated partial volume effect correction increases the statistical power for detection of ^18^F-florbetaben uptake in aging APP-Swe mice by 10 % [15].

## Florbetaben pharmacokinetics

Results from clinical pharmacology studies demonstrate that, after intravenous bolus injection of 300 MBq florbetaben, an ^18^F-radioactivity concentration of 2–3 % injected dose/L was achieved in arterial plasma 10 min post-injection (p.i.). Brain radioactivity uptake was rapid, reaching a maximum of ~6 % of injected ^18^F-radioactivity at 10 min p.i. Florbetaben was eliminated from the plasma of patients with AD dementia and healthy controls (HCs; primarily via the hepatobiliary system) with a mean biological half-life of ~1 h. No relevant radioactivity was measured in blood at 4 h p.i. [16].

To investigate the influence of ethnicity on the florbetaben PK, two clinical studies with identical designs were performed in healthy Caucasian and Japanese volunteers [16]. Among the secondary objectives were the comparative evaluation of PK and florbetaben metabolism, based on a single administration of 300 MBq florbetaben, with either a low (≤5 µg) or high (50–55 µg) tracer mass dose. PK parameters were evaluated based on the total ^18^F-radioactivity in plasma and urine, followed by metabolite analysis using radio-high-pressure liquid chromatography. Both ethnic and tracer mass dose differences were evaluated. Values for the calculated dose-normalized area under the concentration–time curve from time 0 to last measurement (AUC_0–tlast_) were similar between the low and high doses, as well as between Caucasian and Japanese individuals. Furthermore, no significant differences in AUC_0–tlast_ could be detected between the two populations. Taken together, no differences in the florbetaben PK were detected between Japanese and Caucasians [16].

## Florbetaben dosimetry

^18^F has a radioactive half-life of 110 min. At 12 and 24 h p.i., 98.93 and 99.99 % of the activity, respectively, are physically decayed. Detailed doses for all organs were assessed for 17 Caucasian healthy volunteers with a mean body weight of 73.8 kg, and for 18 Japanese healthy volunteers with a mean body weight of 58.8 kg. Dosimetry calculations were adapted to the adult model for the Caucasians (with a body weight of 73.7 kg) or to the 15-year-old model for the Japanese (body weight 56.8 kg), calculated using OLINDA (Organ Level INternal Dose Assessment) software.

For ^18^F-florbetaben, the mean effective dose solely (without CT exposure in case of combined PET/CT) resulting from the administration of 300 MBq to Caucasian individuals was 5.8 ± 0.42 mSv. For the Japanese individuals of lower body weight, the mean effective dose was slightly higher (8.1 ± 0.48 mSv), but still well within the range of other radiopharmaceutical diagnostic agents [17].

## Florbetaben safety

The data submission to the health authorities comprised safety assessments from 978 administrations to 872 subjects who received florbetaben and 12 who received vehicle. The results show that florbetaben at the recommended radioactive exposure of 300 ± 20 % MBq and tracer mass doses of up to 55 µg per injection is generally safe and well tolerated. As to be expected with a PET tracer such as florbetaben, no signs of organ toxicity or impaired tolerance resulting from pharmacodynamic or pharmacological drug activity were observed. No significant trends indicative of a safety concern were apparent overall or in subgroup analyses according to sex, age, region or estimated glomerular filtration rate. Furthermore, there was no specific profile for adverse events after the florbetaben PET application. The most common side effects observed in clinical trials were injection-site reaction and injection-site pain [18].

### Comparison with [^11^C]PiB and test–retest variability of florbetaben

**Fig. 2:**
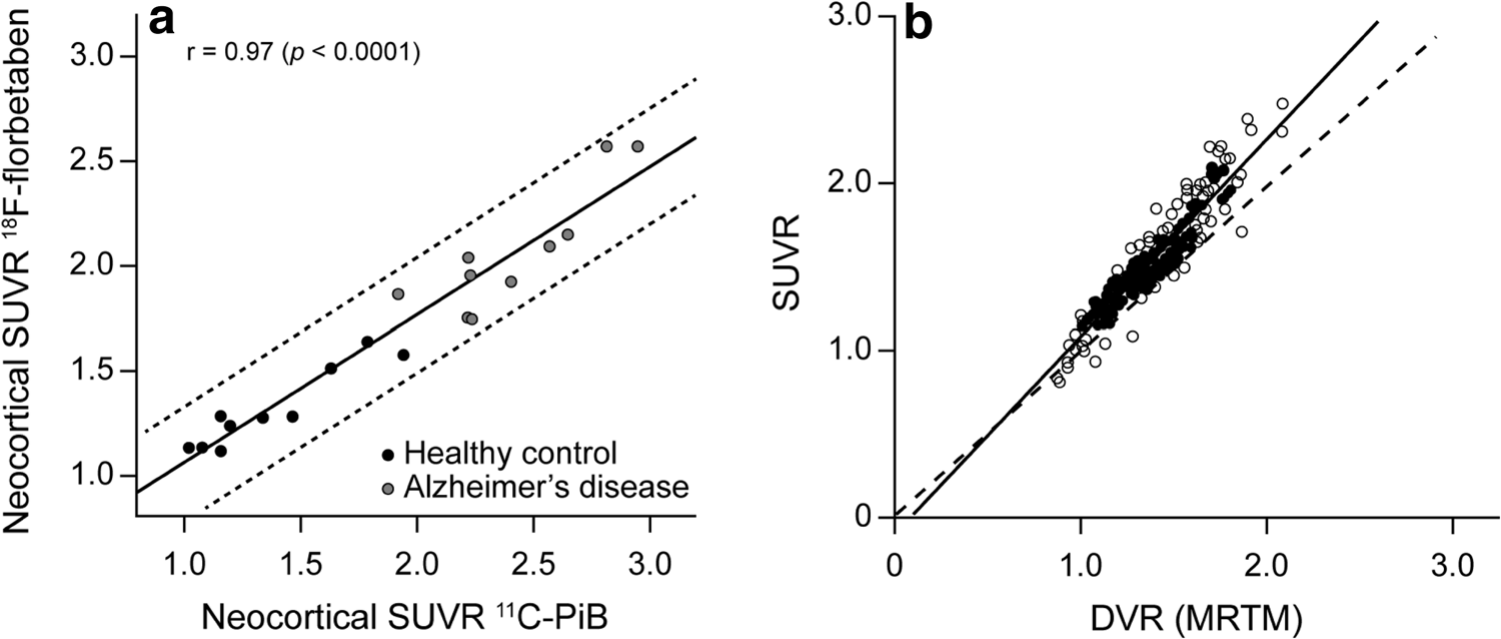
^18^F-florbetaben binding can be accurately quantified by PET imaging. Intra-individual comparison between ^18^F-florbetaben and the gold standard amyloid PET tracer (^11^C-PIB). Standardized uptake value ratios (SUVRs) showing close positive, linear correlation (**a**), and between ^18^F-florbetaben SUVRs and distribution volume ratios (DVRs) as obtained by the gold standard method to quantify PET tracer binding (full kinetic modeling, in this case performed using the multilinear reference tissue model [MRTM]), again showing a close positive linear correlation (**b**). **a** Reproduced from Ref. [19] ©2013 BMJ Publishing Group Ltd. **b** This research was originally published in Ref. [24], copyright by the Society of Nuclear Medicine and Molecular Imaging, Inc

[^11^C]PiB was compared with florbetaben in 10 healthy elderly individuals and 10 patients with AD dementia who had undergone PET imaging after intravenous injection of 370 MBq of [^11^C]PiB and 300 MBq of florbetaben under separate research protocols [19]. [^11^C]PiB and florbetaben images were processed and analyzed similarly and co-registered so that placement of regions of interest (ROIs) was identical on both scans. The results showed that standardized uptake value ratios (SUVRs), taking the cerebellum as reference region, were significantly higher (*p* < 0.0001) for both tracers in patients with AD dementia versus health controls in most cortical areas. Global SUVRs in patients with AD dementia were on average 75 % higher than in HCs with PiB and 56 % higher with florbetaben, with an excellent linear correlation between PiB and florbetaben global SUVRs (*r* = 0.97, *p* < 0.0001) (Fig. 2a). Effect sizes for distinguishing AD dementia from HCs were similar for both radiotracers (Cohen’s d: 3.3 for PiB, 3.0 for florbetaben). Further, the test–retest variability of ~6 % was within acceptable limits [20].

### Optimal scan duration for florbetaben

**Fig. 3:**
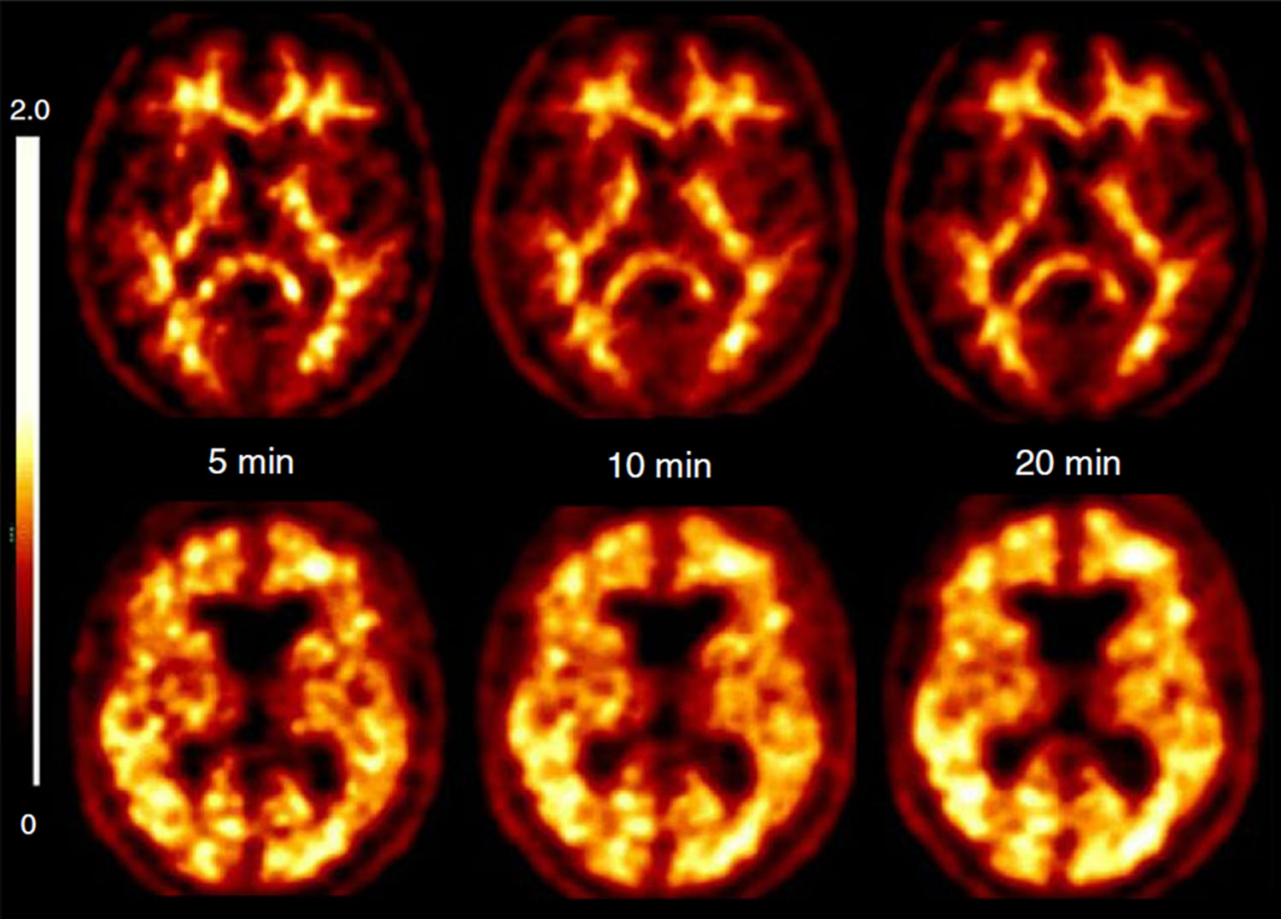
^18^F-florbetaben PET scan duration is flexible. Transverse PET slices of an Alzheimer’s disease patient (*upper row*) and of a healthy control (*lower row*) of different scan durations, all starting 90 min after tracer administration. No relevant loss of image quality and discrimination ability reducing scan time from 20 to 10 min or 5 min. Reprinted from ref. [21], ©2013 BMJ Publishing Group Ltd

The effect of scan duration on the accuracy of florbetaben PET was evaluated using data from 25 patients with AD dementia and 25 healthy volunteers [21]. In each participant, scans of 5, 10 and 20 min duration were analyzed, all starting at 90 min p.i. Visual assessment was conducted by three blinded experts, and the presence or absence of β-amyloid, and diagnostic confidence (0–100 %) were scored. Randomly selected datasets of 10 patients with AD dementia and 10 healthy volunteers were quantified using an established volume of interest (VOI)-based approach and a voxel-based approach. The sensitivity and specificity of the blinded read were each (for three independent blinded readers) 80 and 96 %, respectively, for all scan durations, with diagnostic confidence of 95 ± 8, 97 ± 6 and 97 ± 6 % for the 5-, 10- and 20-min scans, respectively. Inter-reader agreement was high (kappa = 0.89–0.94), and intra-reader agreement was significantly higher for the 20-min scan (kappa = 1.00) than the 5- and 10-min scans (kappa = 0.80 [*p* = 0.003] and 0.71 [*p* = 0.002], respectively). For all scan durations, composite SUVRs (Cohen’s d effect size: 3.9–4.5; all *p* < 0.0001) (Fig. 3) and individual brain volumes affected by β-amyloid (Cohen’s d effect size: 1.6–2.0; all *p* < 0.005) were significantly higher in patients with AD dementia versus HCs. From this study it was concluded that in cases in which the recommended scan duration of 20 min (starting 90 min after tracer administration) is not realistic due to the patient’s inability to cooperate (a feature that might occur quite often, especially in the target population of dementia patients), a reduction of the scan duration is possible for florbetaben without losing diagnostic accuracy [21].

## Florbetaben efficacy evaluation for β-amyloid detection

Throughout a comprehensive phase 0–III clinical development program, the efficacy of florbetaben was established for detecting neuritic β-amyloid plaques by means of PET imaging. These trials employed different standards of truth (SoTs) depending on the clinical scenario. In one scenario, clinical diagnosis of AD dementia served as the SoT, given that β-amyloid plaques are one hallmark of AD. In another instance, clinical diagnosis of Down’s syndrome (DS) was employed as the SoT, given that the brains of patients with DS will, at certain ages, inevitably experience pathological β-amyloid plaque accumulation. In another scenario, progression toward dementia in MCI served as the SoT. Finally, and most importantly, postmortem histopathology for β-amyloid plaques served as the ultimate SoT in the pivotal phase III trial. The following paragraphs summarize the results of these florbetaben efficacy trials.

### Proof-of-concept studies

The first-in-human study evaluating florbetaben (then known as BAY94-9172) for its potential to image neuritic β-amyloid plaques in AD dementia patients was carried out in Australia by Rowe et al. in 2008 [22]. In this study, 15 AD dementia patients and 15 HCs, as well as five patients with frontotemporal lobe degeneration (FTLD), were subjected to florbetaben PET imaging. High discriminative power was observed for both visual and quantitative PET data obtained: all AD dementia patients were scored as positive for Aβ, 13 of the 15 HCs as Aβ-negative, and all FTLD patients as Aβ-negative in the visual analysis. The quantitative analysis was consistent with the visual assessment demonstrating that neocortical tracer uptake was significantly higher for the AD dementia patients compared to HCs (SUVR 2.0 ± 0.3 vs. 1.3 ± 0.2; *p* < 0.0001) [22].

As a follow-up proof-of-concept study in Europe, a similar phase 0 study was carried out in 2008 in Germany. Here, 10 patients with probable AD dementia and 10 age-matched HCs received a single dose of 300 MBq florbetaben. PET data at 70–90 min p.i. were analyzed visually by three blinded experts, with quantitative assessment performed via magnetic resonance imaging (MRI)-based anatomical sampling of predefined VOIs and calculation of SUVRs [23]. Individual “whole-brain β-amyloid load” was calculated by single-case voxelwise analysis. Based on visual PET analysis, 9/10 patients with AD dementia, but only 1/10 HCs, were β-amyloid positive (*p* = 0.001). Inter-reader agreement between the three experts was high (weighted kappa ≥ 0.88). Neocortical SUVRs were significantly higher in patients with AD dementia than in HCs; effect size was largest in the frontal cortex and smallest in the parietal cortex (*p* = 0.003–0.010) and was confirmed by voxel-based group comparison.

In the above trial, full kinetic modeling, including dynamic PET imaging and arterial blood sampling, was employed to fully characterize the [^18^F]florbetaben pharmacokinetics in the brain [24]. Regional brain-tissue time–activity curves for 90 min were analyzed by one- and two-tissue-compartment (1TCM and 2TCM) models, with metabolite-corrected plasma data estimating the specific distribution volume (VS) and distribution volume ratio (DVR). DVR was also estimated using a multilinear reference tissue model (MRTM) with cerebellar cortex as the reference tissue. SUVRs were calculated at 70–90 min p.i. Results showed that the time–activity curves for all brain regions were best described by 2TCM. β-amyloid-binding parameters in the cerebral cortex were significantly increased in patients with AD dementia (*p* < 0.05), and there were significant linear correlations among these parameters (*r* ^2^ = 0.83). Effect sizes in group discrimination between the β-amyloid-positive AD dementia scans and the β-amyloid-negative HC scans for all binding parameters were largest for DVR (2TCM) (4.22) and smallest for VS (3.25), and intermediate and the same for DVR (MRTM) and SUVR (4.03). Mean SUVRs (1.52 ± 0.32) were 12 % higher than the mean DVR (MRTM) values (1.36 ± 0.25), and there was also a strong linear relationship between the SUVRs and DVR (MRTM) values (*r* ^2^ = 0.90, *p* < 0.0001; Fig. 2b). These data provide the rationale for using SUVRs in future clinical routine application of [^18^F]florbetaben as a quantitative β-amyloid plaque readout, showing only a small bias toward overestimating β-amyloid binding.

### Florbetaben to assist in the diagnostic evaluation of patients with different dementing diseases

**Fig. 4:**
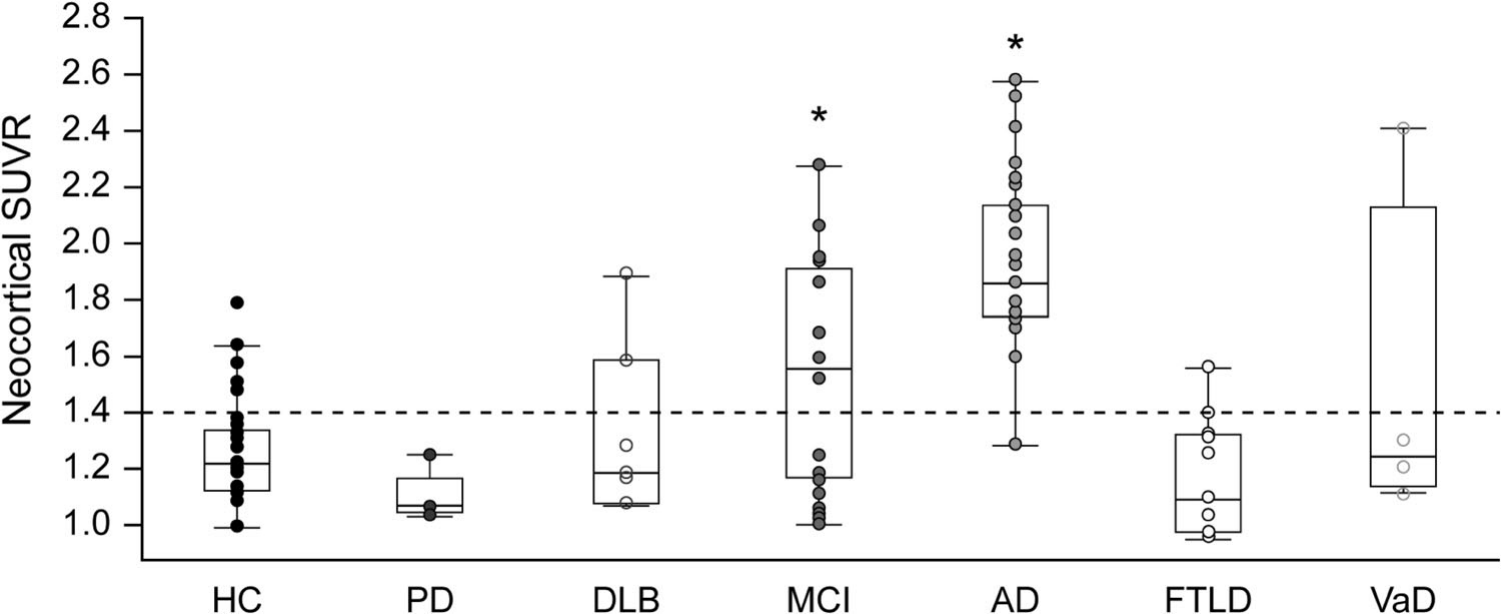
^18^F-florbetaben binding differs between different dementia types. Varying composite neocortical standardized uptake value ratios (SUVRs) in groups of healthy controls (HC), patients with Parkinson’s disease (PD), dementia with Levy bodies (DLB), mild cognitive impairment (MCI), Alzheimer’s disease dementia (AD), frontotemporal lobar degeneration (FTLD), and vascular dementia (VaD). This research was originally published in ref. [25], copyright by the Society of Nuclear Medicine and Molecular Imaging, Inc

This study was carried out to investigate the performance of florbetaben in different dementia entities. Cortical deposition of β-amyloid assessed by florbetaben PET was compared in 32 HCs, 20 individuals with MCI, 30 patients with AD dementia, 11 with FTLD, seven with DLB, five with Parkinson’s disease (PD), and four with vascular dementia (VaD) [25]. All participants underwent PET between 90 and 110 min p.i. after intravenous injection of 300 MBq of florbetaben, and SUVRs were calculated using the cerebellar cortex as a reference region. Overall, patients with AD dementia had significantly higher neocortical SUVRs compared with the other groups (all *p* < 0.0001; Fig. 4). Almost all patients with AD dementia (96 %) and 60 % of those with MCI showed diffuse cortical florbetaben retention, compared with 9 % of patients with FTLD, 25 % of those with VaD, 29 % of those with DLB, and 16 % of HCs. No patient with PD showed cortical binding [25]. The data of differential probability and degree of florbetaben binding in different dementia entities are in accordance with postmortem β-amyloid histopathology and in vivo [^11^C]PIB PET imaging data in different dementia forms, and suggest that florbetaben PET might have a future role for differential dementia diagnosis.

### Phase II studies using clinical AD dementia diagnosis as SoT

The phase II study was a multicenter, open-label, non-randomized, clinical study in patients with probable AD dementia and healthy volunteers from Europe and the USA [26]. Participants were male and female patients of any ethnicity with the features of probable AD dementia, and cognitively non-impaired HCs. The clinical diagnosis served as the SoT in this study; importantly, pathology examinations have shown that clinical diagnosis can be wrong (i.e., individuals diagnosed with AD dementia who do not show β-amyloid plaques upon autopsy) in 10–30 % of cases [4]. This can lead to false-negatives compromising the sensitivity estimation.

The phase II study consisted of two parts: an exploratory part A (*n* = 150) and a confirmatory part B (*n* = 272). The main purpose of Part A was to assess and refine PET scan acquisition and assessment techniques, compare different imaging time points, develop a visual assessment algorithm, and develop a quantitative assessment tool (an operator-independent, MRI-segmented, automated template for SUVR generation) [26]. Clinical diagnosis was established by the on-site investigator. In this trial, florbetaben PET imaging was carried out in 18 centers in Australia, Germany, Switzerland, and the USA. Altogether, 81 patients with mild-to-moderate probable AD dementia and 69 age-matched HCs were included. Florbetaben uptake in neocortical regions was visually scored by three independent blinded readers, and quantified using SUVRs determined from pre-established VOIs on the individual gray-matter-segmented PET/MRI data.

**Fig. 5:**
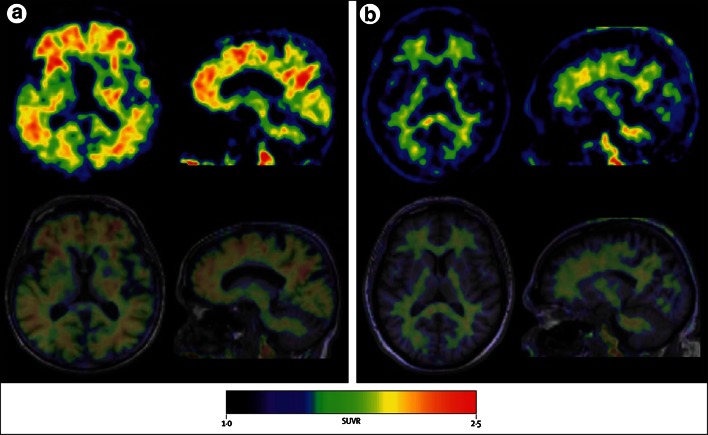
^18^F**-**florbetaben PET detects Alzheimer’s disease with high sensitivity and specificity. PET images (*upper row*) and PET/MRI overlay (*lower row*) of two paradigmatic cases: **a** a patient with Alzheimer’s disease dementia, **b** a healthy control individual, imaged within a worldwide multicenter trial. While in the healthy control, only unspecific uptake in the white matter was observed, this extended to the outer gray matter in the Alzheimer’s disease dementia patient. Reprinted from ref. [26], Copyright (2011) with permission from Elsevier

The blinded read of the 90- to 110-min p.i. florbetaben PET data resulted in a sensitivity of 80 % (95 % confidence interval (CI) 71–89 %) and a specificity of 91 % (95 % CI 84–98 %) in discriminating between the two subject cohorts, which was achieved with high inter-reader agreement (Fig. 5). With regard to the PET image quantification, the SUVRs were significantly higher for the AD dementia patients as compared to the HCs in different neocortical brain regions. Highest effect size for group discrimination was observed for the posterior cingulate (Cohen’s d 1.49). A linear discriminant analysis of the quantitative PET data resulted in a sensitivity of 85 % and a specificity of 91 % for the discrimination between AD dementia patients and HCs. It was also found that in AD dementia patients with Aβ-positive PET scans (according to blinded read), APOE ε4 alleles were more frequent than in Aβ-negative AD dementia patients. A similar tendency was observed for the HCs. During this trial, no safety or tolerability problems were observed for florbetaben PET imaging [25]. In conclusion, this largest and first global trial on an 18F-labeled Aβ-targeted PET tracer provided in a multicenter, multi-camera setting evidence for the efficacy, safety and biological (tracing β-amyloid plaques) relevance of florbetaben PET. In a population representative of a clinical routine situation, the florbetaben PET data could be reliably assessed on both a visual and quantitative basis, as well as quantified in an objective manner.

As a follow-up to this trial, the (mainly unchanged) design from Part A was applied to Part B of the study (*n* = 272), which had a broadened geographic scope with the inclusion of Japanese patients in addition to those from Europe, the USA, and Australia. As the two main study design differences, the SoT in the phase IIB study was clinical diagnosis established by a panel of experts, and the PET images were visually scored by five non-Aβ-PET-expert nuclear physicians after running a web-based training program. PET data of 116 patients with probable AD dementia (age ≥55 years, mini-mental state examination score 18–26, clinical dementia rating 0.5–2) and of 120 cognitively normal elderly volunteers were scored. For the five blinded readers, sensitivity for discrimination between AD dementia patients and healthy volunteers ranged from 69.0 % (95 % CI 60.6–77.4 %) to 81.0 % (95 % CI 73.9–88.2 %), with a median value of 78.5 %, and specificity ranged from 81.7 % (95 % CI 74.7–88.6 %) to 95 % (95 % CI 91.1–98.9 %), with a median value of 89.2 %. Inter-reader agreement was substantial to almost perfect with a kappa of 0.81 (95 % CI 0.77–0.86) across the five readers. These results also led to the conclusion that the training procedure developed leads to accurate and reproducible visual assessments of florbetaben PET data [27].

In conclusion, the two clinical phase II studies confirmed the initial efficacy findings of the phase 0 studies in a larger study population including individuals of varying age and different race. Based on the results, technical parameters could be defined for the phase III program: Part A results were the basis for a re-definition of the rules for the dichotomized decision, for a positive PET scan result (“abnormal”) or a negative PET scan result (“normal”), corresponding to β-amyloid present “yes” or “no”. The Part B results helped to identify potential bias risks for the visual assessment algorithm. Consequently, a refined training program, including the development of an electronic training tool, was developed for future use and for further assessment in the phase III program.

### Phase II study in individuals with Down’s syndrome

**Fig. 6:**
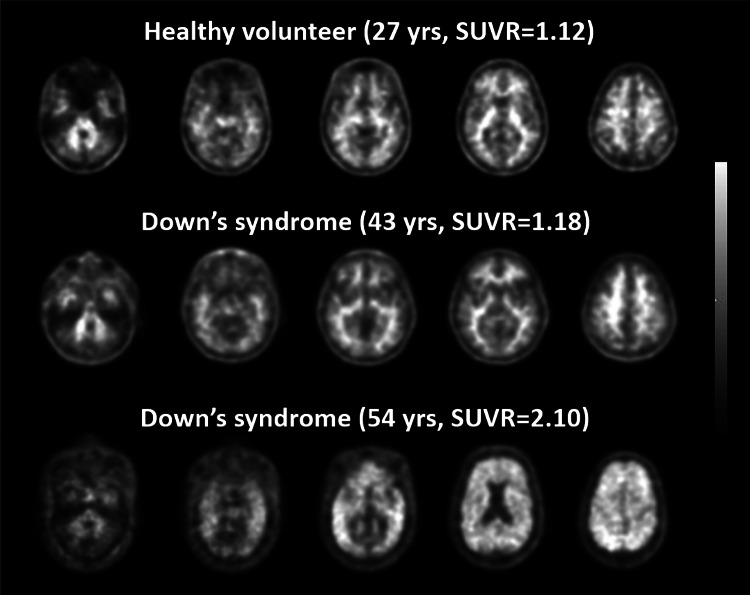
^18^F-florbetaben PET is positive in age-dependent fashion in Down’s syndrome. Transverse PET slices of a β-amyloid plaque-negative healthy control individual (*upper row*), together with those of a 43-year-old patient with Down’s syndrome who was scored as β-amyloid plaque-negative (*middle row*), and those of a 54-year-old β-amyloid plaque-positive Down’s syndrome patient (*lower row*)

The objective of this study was to investigate brain β-amyloid binding in 39 individuals with DS (mean age 46.3 ± 4.7 years) using ^18^F-florbetaben PET imaging [28]. Three blinded independent readers assessed the scans to provide a visual analysis. The primary quantitative imaging outcome was SUVR for six brain regions. Cognitive status was evaluated using the Dementia Screening Questionnaire for Individuals with Intellectual Disabilities (DSQIID). Florbetaben uptake was correlated with age: the percentage of scans visually assessed as positive was 90 % in participants aged ≥50 years (SUVR 1.62 ± 0.26); 53 % in those aged 45–49 years (SUVR 1.43 ± 0.16); and 7 % in those aged 40–45 years (SUVR 1.27 ± 0.11) (Fig. 6). Mean DSQIID score was 6.2 ± 8.0, and there was a significant relationship between β-amyloid and subtle cognitive changes (DSQIID scores > 0). Visual and quantitative assessments were highly correlated (*χ* ^2^ = 11.3823; *p* = 0.0007; Cohen’s kappa 0.58) [28]. In conclusion, brain β-amyloid binding, as measured by ^18^F-florbetaben, increases with age in DS. Individuals with DS and no evidence of dementia demonstrate brain β-amyloid binding in vivo, suggesting that ^18^F-florbetaben PET imaging may detect β-amyloid in this at-risk population.

### Pivotal phase III postmortem histopathology study

In October 2008, the clinical usefulness of β-amyloid-targeted PET tracers for detection of pathological states was verified during a US Food and Drug Administration (FDA) Central Nervous System Advisory Committee meeting, and subsequently confirmed by the FDA. During this process, it was also concluded that, for PET tracers of this type, postmortem histopathology was the most appropriate SoT to obtain regulatory approval for an indication such as “detection of neuritic β-amyloid deposition in the brain.”

Given this regulatory authority view, a phase III study was initiated in 2009 for florbetaben designed as an open-label, non-randomized study to evaluate the efficacy and safety of florbetaben PET imaging for the detection or exclusion of cerebral β-amyloid using postmortem brain histopathology as the SOT. Individuals with a low probability of cerebral β-amyloid deposition (e.g., volunteers without dementia) and individuals with a high probability of β-amyloid deposition (e.g., diagnosis of AD dementia or DLB) were included in the trial. Determination of the presence or absence of florbetaben uptake in the PET scan was compared with postmortem histopathology, and was enriched with PET scan results from healthy volunteers who served as additional negative controls. The study was conducted at 15 study centers: one in Australia, two each in France and Germany, three in Japan, and seven in the USA. Of the 253 subjects screened for study participation, 216 received florbetaben.

To provide the strongest possible level of evidence, the phase III study was designed to demonstrate that florbetaben PET imaging during life can detect the presence or absence of β-amyloid aggregates in precisely the same location as shown in the postmortem histopathological examination. This study provided a more rigorous design to demonstrate that florbetaben PET imaging identifies β-amyloid deposition precisely where noted in the brain by pathology (“target validation”) compared with other evaluations which used global assessments by the readers compared with global assessment by pathology. In this latter design, it is possible to have discordance between the read and the SOT on a regional basis, but agreement globally. It is important to emphasize that the regional tissue-matched florbetaben scan reading method designed for this endpoint with the consensus panel histopathology assessment was not primarily intended to reflect or be used in clinical practice. It was developed to provide evidence that florbetaben binds specifically to β-amyloid plaques, proven by consensus panel histopathological assessment of almost identical tissue-matched regions.

For the regional tissue-matched assessment, photodocumentation of each postmortem brain specimen was used to identify the same region on in vivo MRI images, and finally for co-registration of those with in vivo florbetaben PET images. A rectangular ROI of the matched size and location of the tissue samples used for pathology assessment was then placed on the co-registered PET images. This ensured that the regions visually assessed on the florbetaben scans corresponded almost exactly with the size and location of the tissue samples assessed by the pathologists during autopsy. Readers were instructed to visually assess only the florbetaben gray-matter uptake pattern within the rectangular tissue-matched ROIs. The scan slices containing the rectangular tissue-matched ROIs were presented to the blinded readers in a random sequence, both across brains and across regions (i.e., the six regions of one patient were not presented together, but all regions across all patients were presented to the readers in a random fashion to diminish potential bias). Thus, no assignment of a region to an individual brain was possible. Readers were required to classify the florbetaben gray-matter uptake pattern within each of these small rectangular ROIs in a binary fashion: present (“yes”) or absent (“no”). Readers were extensively trained on the rules for this decision before the reading sessions. A “positive” scan (abnormal; uptake “yes”) should have a gray-matter uptake equal to or higher than the adjacent non-specific white-matter uptake. A PET scan was scored “negative” (normal; uptake “no”) if uptake in the gray matter was lower than the adjacent white-matter uptake.

The six regions chosen for the matched scan and histopathology included regions known to show high and low β-amyloid burden within the same individual: middle frontal gyrus, striatal and parastriatal areas of the occipital cortex, anterior cingulate cortex, hippocampus, posterior cingulate–precuneus, and cerebellum. The postmortem samples of these six regions were examined for the presence of neuritic and diffuse β-amyloid plaques using a combination of modified Bielschowsky silver staining and immunohistochemistry. For the primary efficacy analysis, 31 deceased individuals and 10 young healthy volunteers were evaluated (41 individuals × 6 regions per individual = 246 brain regions; two regions were not evaluable for technical reasons, yielding 244 evaluable regions). The 10 healthy volunteers were included because the high age of the end-of-life patients led to difficulties in acquiring a sufficient number of end-of-life β-amyloid-negative individuals for the specificity cohort. It is important to note that this methodology was developed and applied only for this regional tissue-matched analysis as a target validation study, and was not applied in a subsequent clinically applicable whole-brain visual assessment.

The primary endpoint demonstrated a high correlation between florbetaben accumulation and presence of β-amyloid in multiple regions of the brain in which matched comparison was performed. A high level of inter-reader agreement was achieved for all brain ROIs (kappa = 0.66). Of 106 ROIs, 82 were read as true PET positive, and 130 of 138 ROIs as true PET negative. In areas known to show β-amyloid plaques more frequently in AD (frontal cortex, anterior cingulate, posterior cingulate/precuneus area), tissue-matched ROI sensitivity was 82–90 % and specificity was 86–95 %. Sensitivity was lower (57 %) but specificity higher (100 %) for the hippocampus, resulting in an overall sensitivity of 77.4 % (95 % CI 65.3–89.4 %) and a specificity of 94.2 % (95 % CI 88.6–99.8 %) for all ROIs. In the tissue-matched ROI quantitative florbetaben PET assessment, significantly higher SUVRs were found for regions confirmed to have histopathological evidence of β-amyloid compared with regions that were scored negative for β-amyloid, with the exception of the hippocampus/parahippocampal gyrus, consistent with the visual assessment suggesting that the unique anatomy of this region including potential partial volume errors limits the reliability of PET assessment.

To better reflect future clinical use of florbetaben and similar to other β-amyloid PET histopathology studies, PET scan reads on a subject-level (whole-brain) basis were investigated as a secondary endpoint to determine the sensitivity and specificity of florbetaben in this setting. Results from the visual assessment using the method applicable for clinical use were compared with the neuropathological assessment of the absence/presence of β-amyloid plaques according to the criteria of the Consortium to Establish A Registry for Alzheimer’s Disease (CERAD). This analysis is crucial, as it reflects the expected efficacy of florbetaben in clinical practice. The aim of this part of the phase III study was to evaluate the diagnostic performance using the whole brain visual assessment method proposed for clinical use. This read was done on florbetaben scan images alone without MRI co-registration. After in-person training, three independent readers blinded to clinical and pathology data read all PET images included in the study. The whole brain results of the first 31 deceased individuals and the 10 young healthy volunteers were included in the interim analysis. The visual assessment method designed for this part of the study was subsequently used in studies aiming to assess the efficacy of the florbetaben scan reading by means of an electronic training tool.

In an interim analysis, results of the whole brain assessments included as secondary endpoints were consistent with the overall primary efficacy findings. Visual assessment of florbetaben PET images according to regional cortical tracer uptake (RCTU)/regional cortical tracer binding (RCTB) scoring and brain β-amyloid plaque load (BAPL) scoring compared with the on-site neuropathological assessments according to CERAD (as SoT) showed a sensitivity of 100 % (95 % CI 80.49–100 %), a specificity of 91.67 % (95 % CI 80.61–100 %), and a near-perfect inter-reader agreement (kappa = 0.870).

Results from the interim analysis were subsequently confirmed in a larger cohort and included in EU and US regulatory submissions. The whole brain results of the final analysis, performed on all evaluable brains, were similar to those of the interim analysis. Results for the European Medicines Agency (EMA) and FDA subsets (74 and 82 deceased individuals, respectively, who were available at the respective time points) were similar to the main results. The results from the “EMA set” (*n* = 74), using the majority read of three in-person trained readers compared with onsite neuropathology, are included in the Summary of Product Characteristics (SmPC) (sensitivity: 97.87 [95 % CI 93.75–100 %]; specificity 88.89 (95 % CI 77.03–100 %) [18]. The results from the “FDA set” (*n* = 82), using neuropathology consensus panel-based SoT, are included in the US Prescribing Information. The ranges among the three readers were 96–98 % for sensitivity and 77–83 % for specificity [29]. Moreover, the addition of young healthy volunteers was no longer needed, as a sufficiently high number of β-amyloid-negative individuals became available for the analyses.

### MCI study

Amyloid PET, in the above-described AD biomarker scenario, is considered a valuable tool to increase confidence in understanding the pathophysiological relatedness of MCI and AD [30]. To investigate the potential of florbetaben in predicting AD dementia in MCI subjects, a longitudinal study was designed. In this study, the clinical utility of β-amyloid imaging with florbetaben was assessed in individuals with MCI suspected due to AD and with delayed paragraph recall as an inclusion criterion [31–33]. Florbetaben prognostic accuracy was evaluated for progression to AD dementia by comparing semi-quantitative and visual scan assessments, and by exploring the relationships between β-amyloid, hippocampal volume, and memory over time.

Forty-five individuals with MCI underwent florbetaben PET, MRI, and neuropsychological assessment at baseline and 2 years, with clinical follow-up at 4 years. Positive florbetaben scans, defined by a cortical-to-cerebellar cortex uptake ratio (SUVR) ≥1.45, were compared with visual assessment by five readers. Amnestic MCI (aMCI) was defined by a composite episodic memory (EM) *Z* score below −1.5.

**Fig. 7:**
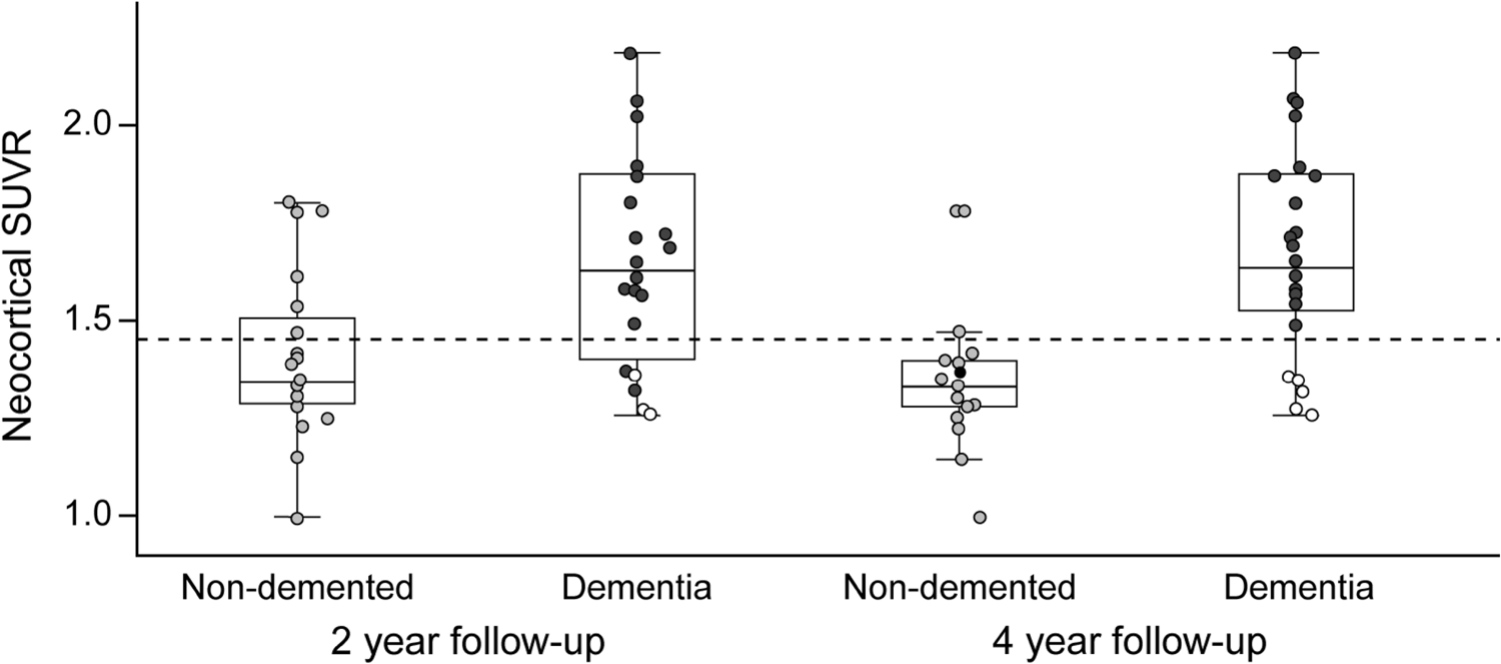
^18^F-florbetaben PET predicts outcome in mild cognitive impairment (MCI). Composite neocortical standardized uptake value ratios (SUVRs) in individuals with MCI who, at the 2-year (*left*) or 4-year (*right*) clinical follow-up progressed to dementia (*dark*-*gray circles* Alzheimer’s disease, *white circles* non-Alzheimer’s dementia) in comparison to those who remained non-demented (*light*-*gray circles*). High predictive power of ^18^F-florbetaben PET was calculated. Reproduced from ref. [33], with permission from BMJ Publishing Group Ltd

At baseline, 24 (53 %) individuals with MCI were positive for florbetaben PET [31]. Majority reads agreed with SUVR classification (kappa = 0.96). After 2 years, 18 (75 %) florbetaben PET-positive individuals had progressed to AD dementia compared with two (9.5 %) florbetaben PET-negative individuals, yielding a predictive accuracy of 83 % [95 % CI 61–94 %] [32] (Fig. 7). Four florbetaben PET-negative individuals developed non-AD dementia. Predictive accuracies of hippocampal volume (58 % [95 % CI 42–73 %]) and aMCI status (73 % [95 % CI 58–81 %]) were lower, and combinations did not improve accuracy. At 4 years, 21 (87.5 %) florbetaben PET-positive individuals had AD dementia whereas five (24 %) florbetaben PET-negative individuals had non-AD dementia, yielding a predictive accuracy of 94 % (95 % CI 74–99 %). While the strong baseline association between florbetaben SUVR and EM declined over 2 years, the association between EM and hippocampal volume became stronger. Florbetaben SUVR increased by 2.2 %/year in florbetaben PET-positive individuals, with no change in florbetaben PET-negative individuals. In conclusion, ^18^F-florbetaben β-amyloid imaging facilitates accurate detection of prodromal AD. As neurodegeneration progresses, and in contrast with the early stages of the disease, hippocampal atrophy—not β-amyloid—seems to drive memory decline.

## Florbetaben PET image acquisition and analysis

**Fig. 8:**
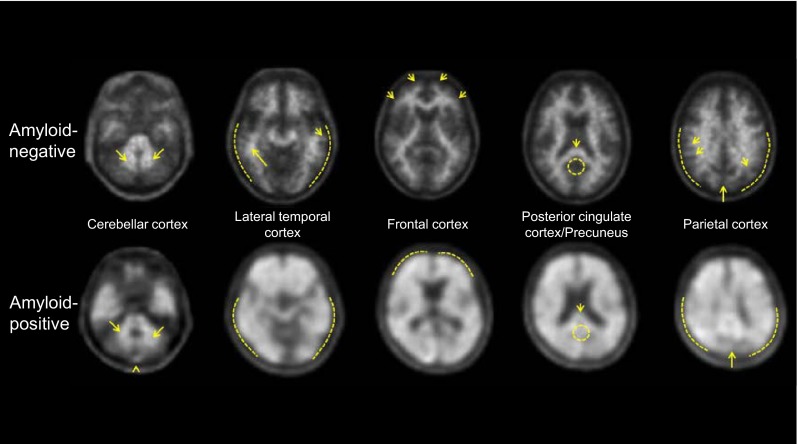
^18^F-florbetaben PET images are easy to interpret visually by trained readers. Typical transverse PET slices at different brain levels of an individual judged as negative for β-amyloid plaques (*upper row*) and of an individual judged as positive for β-amyloid plaques (*lower row*). Cerebellar cortex serves as a reference region from which, step-by-step, the evaluation of the four main target regions (lateral temporal cortex, frontal cortex, posterior cingulate cortex/precuneus; and parietal cortex) starts. A target brain region is scored positive for β-amyloid plaques when tracer uptakes is not observed only in white matter (*arrows*), but also expands toward the outer gray matter (*dashed lines*)

In future clinical routine use of florbetaben, potential readers will need to undergo an a priori training program. For that purpose, a web-based florbetaben reader training tool was developed. Figure 8 demonstrates key features that are communicated in this tool to future florbetaben readers.

A recent investigation assessed the reproducibility (inter- and intra-reader agreement) of the visual assessment of PET scans after completing this web-based florbetaben reader training program from a patient population that closely represents the future clinical use population. ^18^F-florbetaben PET scans were pooled from clinical studies and randomly assigned for consecutive, blinded visual assessment by five independent blinded nuclear medicine physicians. In total, 461 ^18^F-florbetaben injections from phase I–III clinical trials were included in the analysis. Images were acquired from patients with diagnoses of probable and possible AD dementia (mild to moderate), other dementia subtypes, and MCI, and from healthy volunteers. Readers had limited or no prior experience with β-amyloid PET visual interpretation and were trained on an electronic training tool; 10 % of images were randomly re-presented to readers for the assessment of intra-reader agreement. The primary endpoint showed a kappa value of 0.787 (95 % CI 0.750–0.824) across all five readers. The inter-reader agreement was high in all reader pairs, ranging from 0.677 (95 % CI 0.609–0.744) to 0.865 (95 % CI 0.819–0.911). In conclusion, a computer-based web training tool provides high inter- and intra-rater reliability and efficacy in the visual assessment of ^18^F-florbetaben PET among inexperienced readers of scans from a clinically relevant patient cohort.

General recommendations on how to acquire and analyze florbetaben and other β-amyloid tracer PET images will soon be published as Amyloid PET Procedure Guidelines jointly developed by the Society of Nuclear Medicine and the European Association of Nuclear Medicine.

As specified in the EU SmPC, ^18^F-florbetaben PET images are read in a transaxial orientation using a gray scale [18]. The reader should compare the cortical gray-matter signal intensity to the maximum white-matter signal intensity in a systematic manner, starting at the cerebellum and scrolling up through the lateral temporal and frontal lobes, then to the area of the posterior cingulate cortex and precuneus, and finally to the parietal lobe. Interpretation of the images is made visually comparing the activity in cortical gray matter with activity in adjacent cortical white matter. Each of these brain regions—lateral temporal cortex, frontal cortex, posterior cingulate cortex/precuneus, and parietal cortex—should be systematically visually assessed and scored according to the RCTU and BAPL scores. The RCTU scoring system grades the tracer uptake in each of the above regions as 1 = no binding, 2 = minor binding, and 3 = pronounced binding. The RCTU scores for the frontal cortex, posterior cingulate cortex/precuneus, lateral temporal cortex, and parietal cortex are then condensed into a single three-grade scoring system for each PET scan; the BAPL score: 1 = no β-amyloid load, 2 = minor β-amyloid load, 3 = significant β-amyloid load. BAPL scores of “1” are classified as “β-amyloid-negative PET scan”, and BAPL scores of “2” and “3” as “β-amyloid-positive PET scan” (for more details, see florbetaben SmPC [18]).

## Impact of florbetaben on diagnostic confidence and economic implications

Diagnostic confidence was evaluated as an add-on to the phase IIB study, with a questionnaire given to referring physicians whose patients received florbetaben PET imaging [34]. The questionnaire consisted of six items evaluating confidence in the initial clinical diagnosis before PET results were available, objective results of the florbetaben PET scan, congruency of PET imaging and initial diagnosis, potential change of confidence in the overall diagnosis based on the PET scan, and the importance and characteristics of the potential impact on patient management. In total, 201 questionnaires were completed (probable AD dementia, *n* = 121; controls, *n* = 80). An increase in confidence in the initial diagnosis after PET was reported by 80 % of physicians. Imaging results led to an anticipated impact on intended patient care for 89 % of patients with AD dementia and 35 % of controls [34].

Guo and colleagues investigated the potential economic impact of florbetaben PET in the early diagnosis of AD using a discrete event simulation model [35]. The model allowed exploratory analyses from US payer and societal perspectives, simulating the lifetime course of disease progression and the impact management from initial diagnostic workup to final diagnosis. Model inputs were obtained from a large longitudinal US dataset supplemented with data from public data sources and assumptions. Model results showed that use of florbetaben PET was associated with a substantial decrease in the time to diagnosis. In patients with dementia or pre-dementia, florbetaben PET was the dominant strategy, resulting in cost savings alongside increases in quality-adjusted life years for patients [35].

The results of these two initial studies dealing with the impact of florbetaben PET imaging on patient care and the potential economic impact of this new diagnostic tool are encouraging to further investigate these features, a task of enormous relevance for future reimbursement discussions with healthcare system providers.

## Conclusions and outlook

The evidence presented in this review shows that florbetaben is generally safe and well tolerated. It has a favorable pharmacokinetics and radiation exposure. Further, florbetaben has the potential of predicting development of AD-related dementia in MCI cases and of assisting in the differential dementia diagnosis. Also, it has high diagnostic accuracy, as shown employing different gold standards (from clinical diagnosis to postmortem histopathology), for the detection of neuritic β-amyloid plaques in the brain. A negative florbetaben PET scan can reliably exclude AD. The tracer successfully completed a global multicenter phase 0–III development program and was, as a consequence, recently approved by the US Food and Drug Administration and the European Medicines Agency. Taken together, florbetaben is a valuable β-amyloid-targeting PET tracer with great potential to serve as a biomarker supporting clinical AD diagnosis.

Future developments in the field of β-amyloid PET imaging include the still unsolved role of β-amyloid imaging in the progression to dementia, the presence of β-amyloid in healthy elderly individuals, and the use of (semi)-quantification in the assessment of β-amyloid load. In addition, there may also be a role for florbetaben within a combined PET/MR imaging setup. Here, it is hypothesized that combined β-amyloid PET/MRI has the potential to provide both pathology and neurodegeneration biomarker information in one step, by supplementing clinical testing to increase certainty in MCI and AD dementia diagnosis [36]. Also, the potential of early after-tracer administration florbetaben PET images as [^18^F]fluorodeoxyglucose surrogate is currently being investigated. Further, florbetaben quantification tools are currently being developed for example to increase PET reader confidence in rare ambiguous cases or to facilitate follow-up evaluation after investigational therapeutic interventions.
